# Cryptic diversity in a gastrointestinal acanthocephalan of New World primates from Costa Rica

**DOI:** 10.1038/s41598-023-28585-1

**Published:** 2023-02-10

**Authors:** Ernesto Rojas-Sánchez, Fabián Umaña-Blanco, Ana Jiménez-Rocha, Karen Vega-Benavides, Alejandro Medaglia, Alberto Solano-Barquero, Alicia Rojas, Mauricio Jiménez

**Affiliations:** 1 Hospital de Especies Menores y Silvestres, Universidad Nacional de Costa Rica, Heredia, Costa Rica; 2 Laboratorio de Helmintología, Facultad de Microbiología, Universidad de Costa Rica, San José, Costa Rica; 3 Laboratorio de Parasitología, Escuela de Medicina Veterinaria, Universidad Nacional de Costa Rica, Heredia, Costa Rica; 4 Laboratorio Institucional de Microscopía, Instituto Tecnológico de Costa Rica, Cartago, Costa Rica; 5 Escuela de Biología, Instituto Tecnológico de Costa Rica, Cartago, Costa Rica; 6 Centro de Investigación en Enfermedades Tropicales, Universidad de Costa Rica, San José, Costa Rica

**Keywords:** Parasite biology, Parasite evolution, Infectious-disease diagnostics

## Abstract

*Prosthenorchis elegans* is a worm of the family Archiacanthocephala that infects non-human primates in the Americas, producing an intestinal pathology that may compromise the life of its hosts. Squirrel monkeys, *Saimiri oerstedii citrinellus,* were found with *P. elegans* in Costa Rica. Histopathological analysis revealed a severe pyogranulomatous response composed by macrophages, neutrophils, eosinophils, fibroblasts and lymphocytes. Morphological worm analyses revealed 36 hooks in the proboscis distributed in six rows; and total body, hook and lemnisci length were compatible to the original descriptions of *P. elegans.* In addition, phylogenetic, haplotype network and genetic distance analyses were done on cytochrome oxidase subunit 1, *cox*1, sequences obtained from the collected specimens. Sequences obtained herein clustered separately with high posterior probabilities in a Bayesian Inference tree and showed 8.12% nucleotide differences when compared to *P. elegans* from Colombia. This high divergence was confirmed in the TCS network that separated Colombian and Costa Rican sequences by 32 mutational steps, a genetic distance PCA which separated sequences from both geographical locations by 89.5% and an F_ST_ value of 0.655, indicating the presence of cryptic diversity in *P. elegans*. Additional studies from specimens collected from other definitive hosts and geographical locations are required to better understand the biodiversity of this species.

## Introduction

Agricultural practices, tourism, landscape changes and urbanization have caused forest fragmentation and parasite spillover from wildlife to humans and/or domestic animals during the last decades around the world^1,2^. In Costa Rica, these practices have affected primate populations, considerably threatened their health, and possibly modified parasite transmission to their hosts^3^. It should be noted that wildlife are considered national patrimony in this country, and their conservation and research has been declared of public interest and guarantee by the law^4^. In this regard, *Saimiri oerstedii citrinellus*, the grey-crowned Central American squirrel monkey, is the most endangered primate subspecies of squirrel monkeys from Costa Rica, and most of their threats are consequences of human activities^5,6^. Parasites and other infectious agents represent a risk to the conservation of primates since these can accelerate their population decline and its loss can affect or decrease the population of other related flora and fauna as well^7^.

Most worm species affecting squirrel monkeys have a very low pathogenicity, but in chronic and heavy infections can affect nutrition and predispose to fatal secondary infections^8^. *Saimiri o. citrinellus* from Costa Rica have been reported with different parasites of the phyla Nematoda and Acanthocephala, including *Strongyloides* sp., *Filaroides* sp., and *Prosthenorchis* spp.^6,9–11^. Heavy infections with the acanthocephalan *Prosthenorchis elegans* in captive primates have led to diarrhea, loss of appetite, weakness, intestinal perforations with peritonitis and eventually their death^12,13^. However, contrasting findings of good overall condition in a free-living golden headed lion tamarin (*Leontopithecus chrysomelas*) and Wied´s marmosets (*Callithrix kuhlii*) from Brazil infected with *P. elegans* have been reported. Therefore, the pathogenic potential of *P. elegans* seems to be associated to housing facilities, the potential stress induced in these conditions^14^, or host-specific responses towards infection.

*Prosthenorchis elegans* is a parasitic acanthocephalan of the family Oligacanthorhynchidae that infects the intestinal tract of neotropical non-human primates. The life cycle of this worm involves cockroaches and beetles as intermediate hosts^12^, which are then ingested by vertebrate definitive hosts such as non-human primates^15^. Adult parasites bury their anterior end provided with hooks in the intestinal wall of the terminal ileum, cecum and proximal colon, where they absorb nutrients through their body surface^16^. This induces chronic inflammation and the formation of nodules of 2 to 6 mm characterized by necrosis, and polymorphonuclear and mononuclear cell infiltrates^14,15^. In addition, these nodular formations predispose monkeys to develop enteritis, intussusception, and peritonitis that can eventually lead to their death^15^. Pharmacological treatment is limited. Therefore, primates with high parasite burden and severe clinical manifestations usually require surgery^14,15^. Moreover, the diagnosis of these acanthocephalans relies on the coprological examination of the monkey’s feces, which has very low sensitivity and specificity^17–19^. Since *P. elegans* is a major threat to *S. o. citrinellus* conservation in Costa Rica, the use of molecular methods will greatly improve their detection and our knowledge in this parasite´s transmission dynamics and evolutionary history.

Analysis of the mitochondrial marker, cytochrome oxidase subunit 1 (*cox*1) of *P. elegans* obtained from white footed tamarins (*Saguinus leucopus*) and white-fronted capuchins (*Cebus albifrons*) in Colombia demonstrated the presence of six different haplotypes circulating in these primate populations with up to 1.6% genetic distance between specimens^18^. Interestingly, haplotype segregation in that study did not correspond to host species or holding facility^18^. In contrast, analysis of the *cox*1 and ribosomal markers of the fish acanthocephalan *Leptorhynchoides thecatus* has found a great genetic diversity in the species. Furthermore, patterns of cryptic diversity have been detected in some acanthocephalan lineages which have been explained by host and microhabitat specialization which eventually leads to speciation^20^. In the present work, we explored the morphological and molecular characteristics of *P. elegans* collected from *S. o. citrinellus* in Costa Rica by running morphometric analyses of the specimens and sequencing *cox*1 fragments.

## Results

### Pathological findings

**Figure 1:**
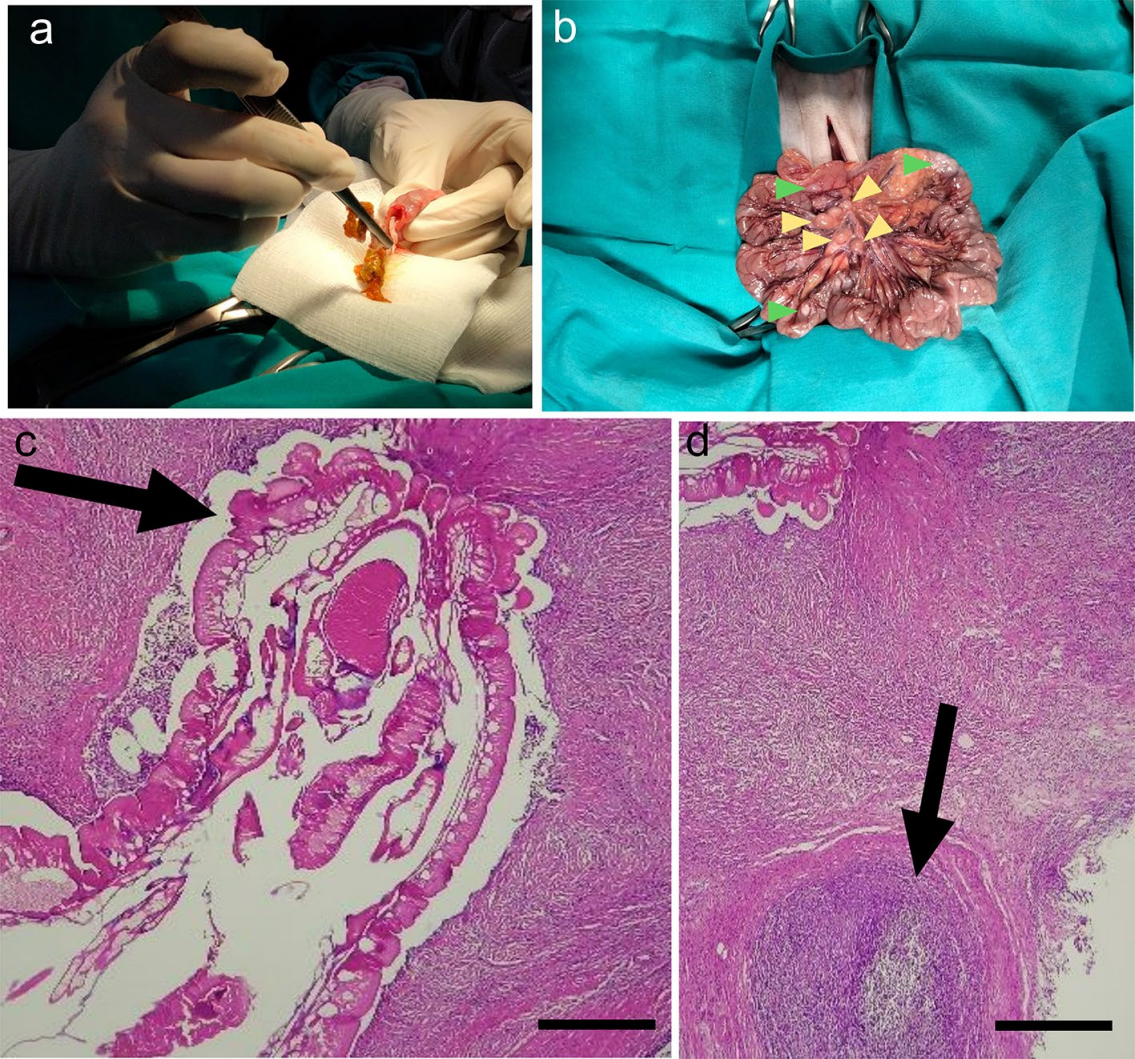
Macroscopic and microscopic findings of intestinal lesions induced by *P. elegans* in *S. o. citrinellus*. (**a**) Extraction of an adult specimen during intestinal surgery. (**b**) Macroscopic appearance of the large bowel showing enlarged lymph nodes (yellow arrowheads) and nodules induced by the worms (green arrowheads). (**c**) Histopathological staining of *P. elegans* worms penetrating the intestinal muscular wall and inducing a severe pyogranulomatous reaction (Bar: 1.5 mm). (**d**) Inflammatory foci in the outer muscle layer and serosa of the intestine consisting of degenerated parasites surrounded by macrophages, neutrophils, eosinophils, fibroblasts and lymphocytes (Bar: 1.7 mm).

Adult *P. elegans* were recovered after surgical gut examination mainly in proximal and terminal ileum, as well as the cecum and on one occasion in the jejunum (Fig. 1a). Intestinal lymph nodes were enlarged and macroscopic nodules with adult acanthocephala were observed (Fig. 1b). Parasites were observed penetrating the intestinal muscular wall (Fig. 1c), causing a severe pyogranulomatous and eosinophilic inflammatory reaction. Moreover, a chronic and transmural pyogranulomatous enteritis with eosinophils was associated with presence of parasites in the serosa tissue (Fig. 1d). The inflammatory foci extended from the lamina propria to the outer muscle layer and the serosa (Fig. 1d). The inflammatory infiltrate was mainly composed of macrophages, neutrophils, eosinophils, fibroblasts and lymphocytes. A large pyogranulomas reaction was associated with the presence of degenerated parasites.

### Morphological analysis of adult worms

**Figure 2:**
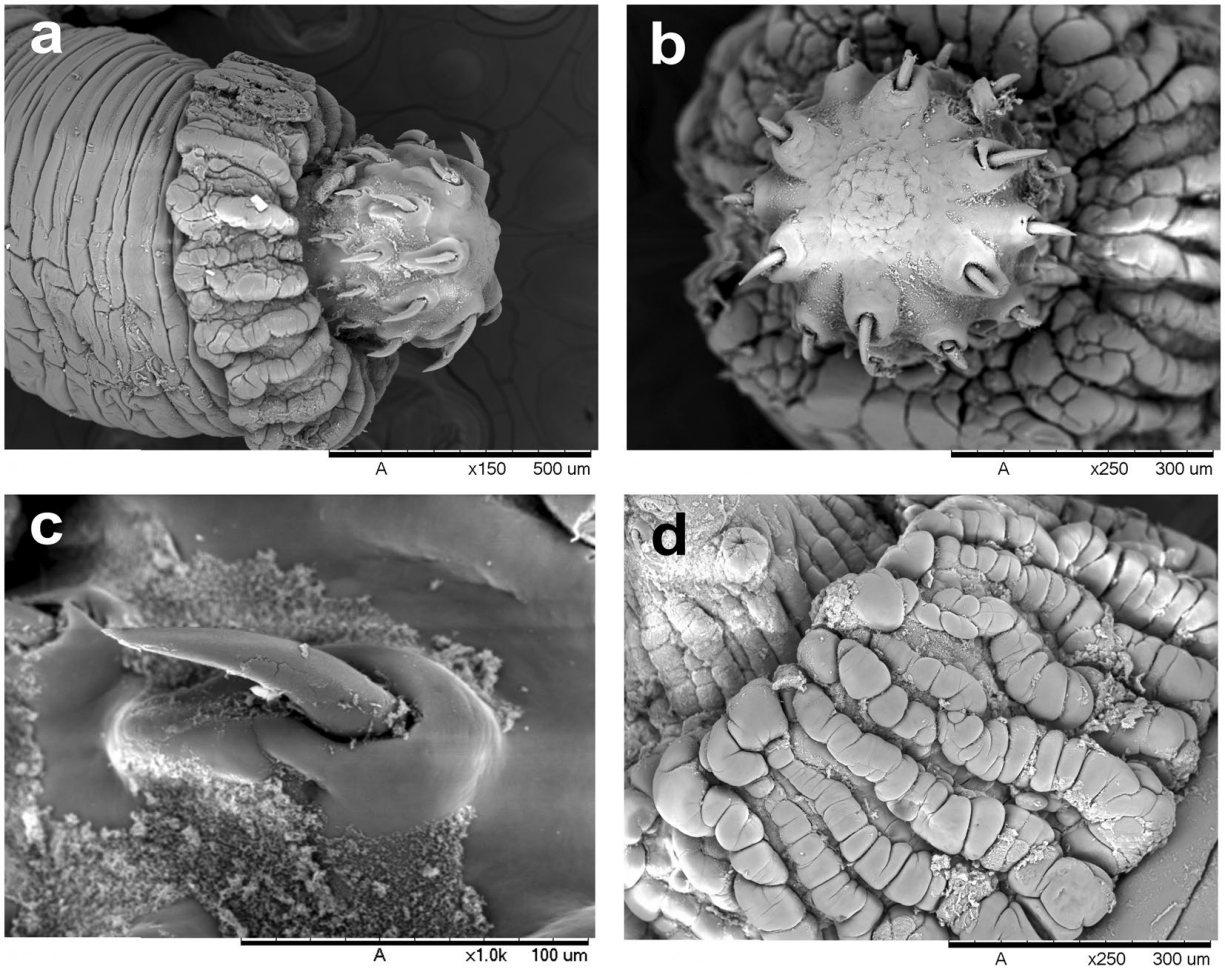
Scanning electron microscopy observations of *P. elegans* adults collected from *S. o. citrinellus*. (**a**) Side view of an adult showing five hook rows in the anterior portion of the body followed by a characteristic collar of the species (Bar: 500 µm). (**b**) Top view of the anterior portion of *P elegans* body. Six hooks per row can be observed (Bar: 300 µm). (**c**) Magnification of a hook (Bar: 100 µm). (**d**) Ribbon characteristic of *P elegans* observed after the protrusible anterior end.

Worms were characterized by the presence of a hooked proboscis (Fig. 2 and Table 1). Males were 23.7 ± 1.75 mm in length and females were 36.4 ± 7.8 mm. Specimens showed a proboscis with six rows of hooks, each with six hooks, for a total of 36 hooks. The length and width of the lemnisci as measured in five specimens were 8.72 ± 0.15 mm and 0.56 ± 0.02 mm, respectively. Hook laminas were measured for the first, second and fifth rows and were 0.12 ± 0.01 mm, 0.09 ± 0.01 and 0.05 ± 0.01, respectively, whereas the hook root was measured for the first row only and was 0.063 ± 0.003 mm. Eggs collected from females were in average 0.064 ± 0.009 mm long by 0.038 ± 0.008 mm wide.

**Table 1:** Morphological measurements of *P. elegans* collected herein compared to the original species description made by Machado-Filho and other *Prosthenorchis *spp. Measurements provided for the specimens of this study correspond to the average with standard deviation.

Character	This study	Prosthenorchis elegans* (Machado-Filho)	Prosthenorchis spirula*	Prosthenorchis confusus*
Total body length in males (mm)	23.7 ± 1.75	23–30	20–30	20–30
Total body length in females (mm)	36.4 ± 7.8 mm	35–50	30–35	25–30
Total number of hooks (rows of hooks, number of hooks per row)	36 (6,6)	36 (6,6)	30 (6,5)	36 (6,6)
Lemnisci shape	Cylindrical and long	Cylindrical and long	Claviform and long	NS
Lemnisci length (range) (mm)	8.72 ± 0.15 (8.53–8.88)	9.85**	5.62–8.19	NS
Lemnisci width (range) (mm)	0.56 ± 0.02 (0.53–0.58)	0.43**	0.236–0.407	NS
Hook lamina (mm)	0.12 ± 0.01 (first row), 0.09 ± 0.01 (second row) and 0.05 ± 0.01 (fifth row)	0.121 (first row), 0.10 (second row), 0.054 (fifth row)	0.189 (first row), 0.159 (second row), 0.058 (fifth row)	0.113 (first row), 0.109 (second row), 0.063 (fifth row)
Hook root (mm)	0.063 ± 0.003	0.021–0.092	0.050–0.176	0.054–0.149
Egg width and length	0.038 ± 0.008 and 0.064 ± 0.009	0.042 and 0.063–0.077	0.042–0.046 and 0.063–0.071	0.052 and 0.078**

NS, not specified.

*The number of individuals for taking each measurement is not specified.

**Not specified if number is an average, minimum, or maximum value.

### DNA analysis

Specimens isolated from squirrel monkeys were 95.04% similar to a *P. elegans* isolate 12-A45-2 from Colombia (GenBank accession number KT818504)^18^. Molecularly analyzed worms belonged to five different monkeys: specimen A was collected from monkey 1, specimen B from monkey 2, specimens C and D from monkey 3, specimens E and F from monkey 4 and specimen G from monkey 5. Sequences generated in this study were deposited in GenBank as *P. elegans* with accession numbers ON458021, ON458022, ON458023, ON458024, ON458025, ON458026 and OQ096471.

**Figure 3:**
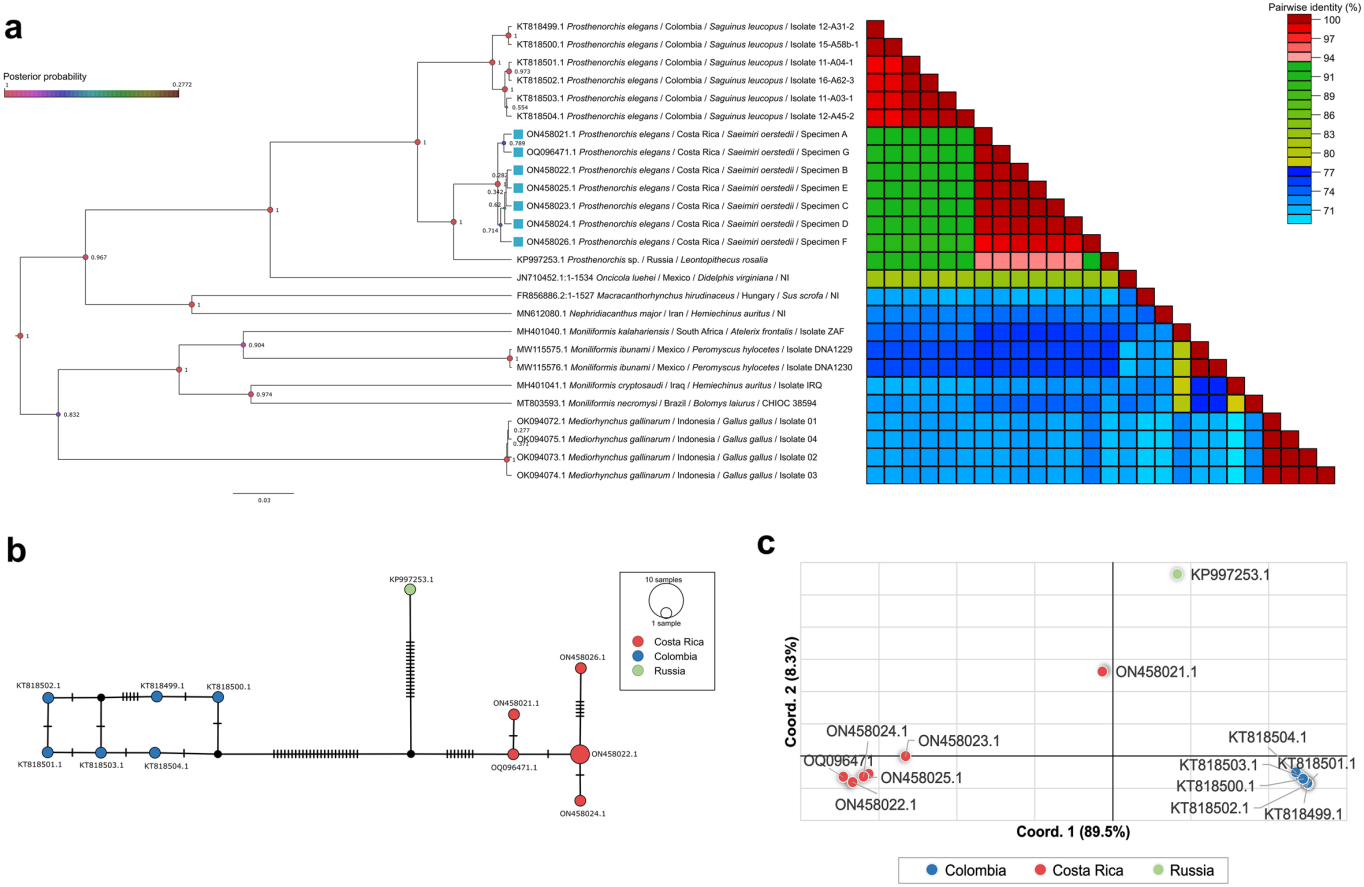
Analysis of a 621 bp fragment of *P. elegans* from Costa Rica and Colombia and *Prosthenorchis* sp. from Russia. (**a**) Bayesian inference phylogenetic tree of *P. elegans* and other Archiacanthocephala worms. Sequences obtained in the present study are marked with a light blue square next to the taxa name. Posterior probabilities are proportional to the node circle size and color scale. The heatmap next to the tree shows the nucleotide p-distances between sequences. (**b**) TCS haplotype network of *P. elegans cox*1 sequences obtained from Costa Rica and Colombia and *Prosthenorchis* sp. from Russia. Circle size is proportional to the number of sequences sharing a haplotype, black circles denote hypothetical haplotypes and hatch marks correspond to mutational steps between sequences. Circles are color-coded according to their geographical location. (**c**) PCA of the genetic distances between *P. elegans* from Costa Rica and Colombia and *Prosthenorchis* sp. from Russia. Circles are color-coded according to their geographical location.

Different acanthocephalan species were grouped in separate clusters with high posterior probabilities as obtained in the Bayesian Inference (BI) phylogenetic tree (Fig. 3a). For instance, *Mediorhynchus gallinarum* isolates obtained from *Gallus gallus* from Indonesia formed a well separated group apart from *Moniliformis* spp., *Nephridiacanthus major* and *Macracanthorhynchus hirudinaceus*. The closest species to *P. elegans* was *Oncicola luehei* obtained from *Didelphis virginiana* of Mexico.

*P. elegans* obtained from Colombian white-footed tamarins (*Saguinus leucopus*) were clearly divided from those collected from *S. o. citrinellus* from Costa Rica and the *Prosthenorchis* sp. collected from Russia with 1.000 posterior probabilities (Fig. 3a). Nucleotide differences within Costa Rican *P. elegans* ranged from 0.36 to 1.72% (mean = 0.59 ± 0.53%) and within Colombian specimens from 0.24 to 1.92% (mean = 1.07 ± 0.7%). In addition, the mean nucleotide differences between specimens from Colombia and Costa Rica was 8.12 ± 0.51%, fluctuating from 7.51 to 10.1%. *Prosthenorchis* sp. from Russia differed from Costa Rican and Colombian *P. elegans* specimens in average by 5.11 ± 0.47% and 9.87 ± 0.28%, respectively. On the other hand, mean nucleotide distances between *P. elegans* from Costa Rica and *O. luehei, M. hirudinaceus, N. major, Moniliformis* spp. and *M. gallinarum* were 17.6 ± 0.22%, 29.1 ± 0.14%, 26.3 ± 0.17%, 26.3 ± 1.46% and 27.7 ± 0.11%, respectively.

The TCS haplotype network showed a clear separation between *cox*1 sequences of *P. elegans* from Costa Rica and Colombia (Fig. 3b), with the Russian *Prosthenorchis* sp. in the middle. Five haplotypes were obtained in the Costa Rican worms, with three specimens belonging to the same haplotype ON458022, whereas the sequences from each Colombian specimen belonged to a separate haplotype. Thirty-three mutational steps separated *P. elegans* from both geographical locations with a hypothetical haplotype connecting each cluster. This deep separation was confirmed in the PCA analysis using the *cox*1 nucleotide distance, with 89.5% of differences explained by the first coordinate that separated the acanthocephalans from Costa Rica and Colombia in two groups (Fig. 3c). The second coordinate explained only 8.3% of the nucleotide differences and further separated Colombian sequences into two subgroups. Finally, the Russian *Prosthenorchis* sp. was separated from specimens from Costa Rica and Colombia.

Genetic distances between Costa Rican and Colombian sequences were also estimated using F_ST_ values. F_ST_ between *P. elegans* from both geographical locations was 0.655 with *p* < 0.00001, suggesting differentiation between both populations due to genetic structure.

## Discussion

In the present study, we report the macroscopic and microscopic pathological findings of *P. elegans* infections in squirrel monkeys from Costa Rica. Furthermore, an in-depth identification process of the collected worms was conducted by performing morphometric observations, scanning electron microscopy and DNA genotyping of specimens. Herein, we have found severe nodular lesions caused by *P. elegans* in monkeys, as well as the cellular infiltrates occurring in intestinal tissues. In addition, the presence of a cryptic clade is suggested given the high nucleotide differences observed in Costa Rican and Colombian specimens as confirmed by phylogenetic, haplotype and PCA analyses.

*Prosthenorchis elegans* has been detected in a variety of non-human primates including golden-headed lion tamarins (*Leontopithecus chrysomelas*) and Wied’s marmosets (*Callithrix kuhlii*) in Brazil^21^, in squirrel monkeys (*Saimiri sciureus*) from Mexico^14^, white-footed tamarins (*Saguinus leucopus*) and white-fronted capuchin (*Cebus albifrons*) in Colombia^18^. Pathology caused by this acanthocephalan is variable and often depends on the underlying immune state of the monkey host due to the stress induced during captivity^15^. For instance, two free-living non-human primate species from Brazil were found with good health condition^21^. However, different studies have reported that captive animals suffer weight loss, and show increased glucocorticoid and neutrophil levels when compared to their wild state^22^. Even though the squirrel monkeys studied herein were free-living, they manifested signs of severe intestinal disease, as has been previously observed in free-ranging marmosets from Brazil that manifested severe chronic transmural ulcerative enteritis^19^. This suggests that other factors such as number of parasites per animal^23^, host species^22^, age, sex or feeding behaviors may influence the course of the infection.

*Prosthenorchis elegans* caused a severe intestinal pathology in wild *S. o. citrinellus* from Costa Rica. An eosinophilic and pyogranulomatous reaction was observed surrounding the worms and extending to the outer muscle layers and serosa. These observations correlate with previous reports that found the worms surrounded by necrotic debris, eosinophils, neutrophils, macrophages, lymphocytes and fibrous connective tissue^10,19,24^. *P. elegans* from Russia were collected from dead captive lemurs and tamarins (*Saguinus oedipus, Saguinus fuscicollis* and *Saguinus midas*) which showed intestinal perforation^25^. On the other hand, free-living tamarins and marmosets from Brazil have not manifested severe intestinal manifestations beyond nodular formations where the *P. elegans* adults are attached^21^. As noted, several primate species are susceptible to infection with this acanthocephalan. Nevertheless, humans have not been reported with *P. elegans* before. However, their susceptibility to *P. elegans* infection has been hypothesized and should not be disregarded^12^.

Morphological observations confirmed the identity of the collected acanthocephalan as *P. elegans* (Table 1). Total body length, number and size of hooks correlated with the descriptions provided by Machado Filho^17^, being total body length in males and females in the lower range than original descriptions, but larger than the observations provided by Catenacci et al.^21^. Even though lemnisci were smaller in size in the specimens collected herein when compared to original *P. elegans*^17^, these were cylindrically-shaped rather than claviform, which are typical of *Prosthenorchis spirula*^17^. Several studies have identified the worms without reporting morphometric analyses^18^. However, these observations should be performed whenever possible, due to the slight morphological similarities between closely related species. For instance, hook number and shape, total body length, size and shape of lemnisci and presence of ribbon in the anterior end should be deeply analyzed^17^. In this sense, molecular assays have facilitated the identification of unknown specimens when their morphological integrity has been damaged or certain structures are difficult to observe to technological limitations^18^. Additionally, DNA-based methods are useful for detecting infections in non-invasive samples, such as feces due to their high specificity and sensitivity^18^.

Molecular analyses of the *cox*1 of *P. elegans* from Costa Rica showed high nucleotide differences when compared to specimens from Colombia, indicating the presence of cryptic diversity in this taxon. Cryptic diversity occurs when specimens are morphologically similar to the type species but are genetically distant, as described for the Phyla Nematoda, Platyhelminthes^26^ and several acanthocephalan species^20,27^. Importantly, the nucleotide distance observed between Colombian and Costa Rican specimens exceeded the expected inter-individual variation since worms were analyzed from independent hosts as supported by the F_ST_ value between these two sequence groups. Furthermore, the F_ST_ indicated a significant divergence between both populations possibly due to genetic structure. This evolutionary process may emerge because of reproductive, ecological, or geographical isolation which eventually leads to heterozygosity and lack of genetic exchangeability within populations^26^. Furthermore, these processes may be affected by climate and environmental alterations that may result to faunal mosaics^28^. Ribosomal and mitochondrial DNA analyses of worms from the class Archiacanthocephala have found a clear clustering across families^29^. The clustering of *Prosthenorchis* sp. from Russia apart from *P. elegans* from Colombia^29^ was confirmed in our study, as well as the six *P. elegans* haplotypes previously found in Colombia^18^. Genetic variability in *P. elegans* may have emerged because of allopatric speciation, since host exploitation does not seem to play a role. Falla et al.^18^ found a low genetic divergence among *P. elegans* collected from two different New World monkey species from Colombia^18^. Therefore, *P. elegans* may be considered a broad generalist that can exploit a wide variety of host species. In this case, geographical separation may have led to genetic drift in these populations, as also hypothesized for the fish acanthocephalan *Leptorhynchoides thecatus*^20^.

Cryptic and genotypic divergence may lead to differences in pathogenicity, as observed in the canid nematode *Onchocerca lupi*^30^. In the present study a severe tissue inflammation was induced by infection with *P. elegans* in squirrel monkeys. However, pathological observations were not reported for *P. elegans* from Colombia^18^ or *Prosthenorchis* sp. from Russia. The latter specimens were collected from captive non-human primates and *Blatella germanica* cockroaches. Therefore, an association between cryptic clades and disease severity cannot be drawn with the current data.

Humans and wild primates have been increasingly sharing habitats due to forest fragmentation as a consequence to their activities^31,32^. Tourism growth and urbanization in Quepos, Puntarenas has come with poor waste management^33^, with the subsequent increase of *P. elegans-*intermediate hosts such as cockroaches. In addition, change in the environment has directed to modifications in fauna dynamics, including loss of native species and increase in the geographical distribution of others^32^. Altogether, this has favored the concentration of pathogens in a reduced number of host species, leading to a more severe pathology. *S.o. citrinellus* is an endangered subspecies that has suffered from anthropogenic activities such as car hits, electrocutions, and habitat fragmentation^33^. As seen here two of these animals from the wild were killed by cars. When bodies were inspected granulomas and parasites were found in the abdomen as described in captivity. This has added another threat to this in situ species’ conservation. Economic resources for conservation are limited by the Government^34^ even when it has the obligation to anticipate, prevent and attack the causes of biodiversity loss^4^. Public policy changes supported by scientific studies should mitigate hosts extinction and are highly relevant for the conservation of this and other endangered species.

## Materials and methods

### Sample collection

**Figure 4:**
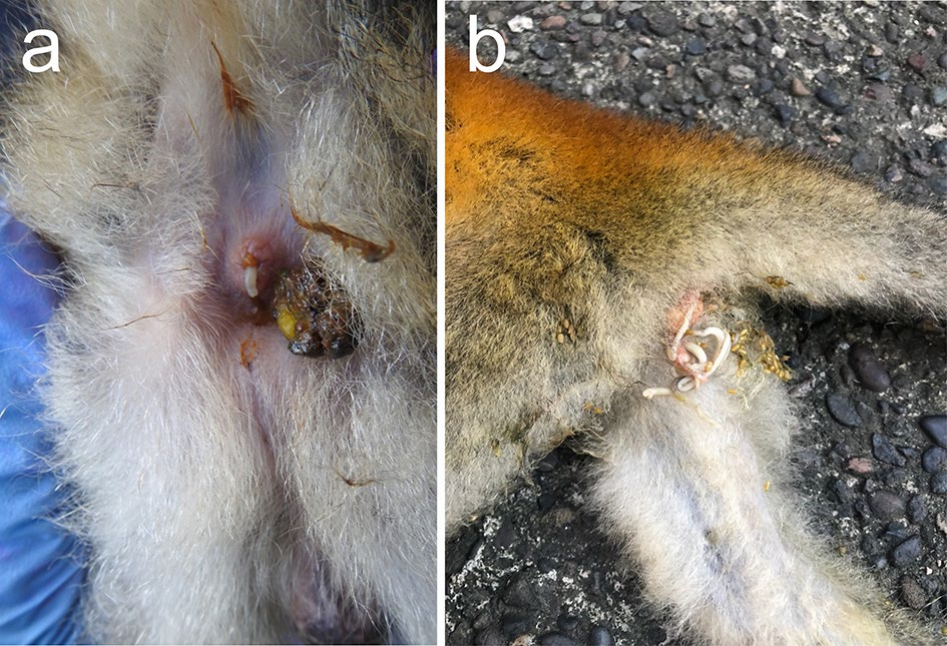
Macroscopic findings of *P. elegans* in squirrel monkeys. Worms expelled from the anus of *S. o. citrinellus* during clinical evaluation.

Parasites were collected from five grey-crowned Central American squirrel monkeys (*S. o. citrinellus*) brought to the Small Animal and Wildlife Hospital, Universidad Nacional (HEMS-UNA) through the wildlife government authority (SINAC). One animal was brought to the hospital for surgical intervention due to nonspecific symptomatology including anorexia, weakness, and progressive weight loss. The second monkey was seen to release naturally the worms from the anus (Fig. 4a), while the other two animals were road-killed by cranial trauma (Fig. 4b). All four animals came from Quepos, Puntarenas, Costa Rica and were sent to the HEMS-UNA for clinical evaluation. A complete clinical evaluation, and ultrasonography tests performed. Two of the monkeys were returned to the place of origin when their health status improved. Intestinal surgery detected adult parasites in the abdominal cavity of the car-hit animals and the one that arrived with clinical manifestations (Fig. 1a). All worms (n = 34) were collected and placed in 70% ethanol for further assays. An intestinal biopsy was obtained from one of the animals for histopathological analyses. This study was done according to the regulations of the National Committee of Biodiversity, CONAGEBIO, and approved in permit R-CM-UNA-005-2021-OT-CONAGEBIO and the regulations of wildlife by the Ministry of Environment and Energy approved in the permit SINAC-PNI-ACOPAC-021-2019.

### Morphometric analysis of specimens

Parasites were sent to the Laboratory of Parasitology of the School of Veterinary Medicine, National University of Costa Rica for their identification. Morphological identification was done following the descriptions by Sokolov et al.^25^. Then, samples were sent to the Laboratory of Helminthology of the University of Costa Rica for genotypic and molecular characterization.

External anatomy of four males and four females wase analyzed by using a digital caliper (to the nearest 0.01 mm in precision). Additionally, five specimens were dissected to examine internal anatomy and lemnisci. Proboscis was mounted for light microscopy observations and characteristics of hooks were annotated as well as their measurements.

### Scanning electron microscopy analysis (SEM)

The anterior and posterior ends of four worms were cut with a sterile blade and processed. SEM was performed at the Institutional Laboratory of Microscopy in the Instituto Tecnológico de Costa Rica. Worms were initially fixed in 2.5% glutaraldehyde and 2% paraformaldehyde in phosphate buffer 0.1 M, pH 7.4 for 48 h. Then, the acanthocephalans were washed with phosphate buffer (0.1 M, pH 7.2) and post-fixed with 1% osmium tetroxide (OsO_4_). Thereafter, worms were washed with distilled water and dehydrated using ethanol. Fixed specimens were dried using a critical point dryer model EM CPD300 (Leica, Wetzlar, Germany) and mounted on aluminum holders with a carbon double-sided adhesive tape. Samples were sputter-coated with gold using an EMS 150R ES sputter coater (Electron Microscopy Sciences, Philadelphia, United States) and observed in a Scanning Electron Microscope model TM-3000 (Hitachi, Tokyo, Japan) at 7.5 kV accelerating voltage.

### DNA extraction and PCR

A 0.5 mm-long piece of the acanthocephalan´s body (n = 17) was cut using a sterile blade and DNA extracted using the Dneasy Blood & Tissue kit (Qiagen, Germany) according to the instructions of the manufacturer. DNA was eluted in 100 µl of elution buffer and stored at − 20 °C for further analysis. After this, an approximately 650 bp fragment of the cytochrome oxidase subunit 1 (*cox*1) was amplified using LCO1479 (5′- GGTCAACAAATCATAAAGATATTGG-3′) and HCO2190 (5′- TAAACTTCAGGGTGACCAAAAAATCA-3′) primers^35^ by denaturing at 95 °C for 5 min, 35 cycles of amplification at 95 °C for 1 min, 54 °C for 1 min and 72 °C for 1 min, followed by a final amplification step at 72 °C for 5 min. Amplicons were visualized in 1.5% agarose gels and sequenced using the BigDye terminator cycle sequencing chemistry (Macrogen, South Korea). The obtained sequences (n = 8) were cleaned, primer sequences were removed and compared to the GenBank database. Species assignment was done when more than 97% of identity to a match was obtained.

### Phylogenetic and haplotype analyses

*Prosthenorchis elegans* and other closely related acanthocephalan species sequences available in GenBank were retrieved and aligned in MEGA7 with the MUSCLE algorithm. The best nucleotide substitution model was calculated in MEGA7 using the Bayesian Inference Criteria. Then, a Bayesian Inference phylogenetic tree was reconstructed using the Bayesian Evolutionary Analysis by Sampling Trees (BEAST) package. First, sequences were uploaded in BEAUTi to generate the .xml file with 10^7^ Markov Chain Montecarlo generations, a sampling frequency of every 10^3^ generated trees and a burnin length of 10^2^ states. The convergence of the chains was verified using Tracer 1.6.0 with values greater than 300 effective sample sizes (ESS) in all priors. The generated trees were converged with TreeAnotator 1.8.4 and visualized using FigTree 1.4.3. Node color and line width was directly proportional to the posterior probabilities.

Nucleotide p-distances were calculated in MEGA7 using the best nucleotide substitution model calculated in the same software. The matrix was uploaded to the Sequence Demarcation Tool (SDT) software and the generated heatmap was proportional to the percentage of similarity between sequences. In addition, a Templeton-Crandall-Sing (TCS) haplotype network was drawn using the PopArt software (available at http://popart.otago.ac.nz) with the statistical parsimony algorithm and a 95% connection limit. A principal coordinate analysis was done using Costa Rican and Colombian *P. elegans cox*1 sequences using the GenAIEx 6.5 software to depict the separation between the two geographical locations. Finally, F_ST_ and their respective *p* values were calculated with the Arlequin software 3.5.2.2 using three sequence groups: Costa Rican, Colombian and Russian^36^.

## Conclusions

The present work focused on the pathological, morphological, and molecular characteristics of *P. elegans* collected from squirrel monkeys in Costa Rica. We found that the specimens studied herein induced a strong pyogranulomatous reaction in the intestine of its hosts, which questions the factors increasing the pathogenicity, that may include worm burden, host immune status and stress. Interestingly, cryptic diversity was found in the *P. elegans* studied herein which separated *cox*1 sequences of Costa Rican specimens from worms of other geographical locations. Additional studies for the identification of the intermediate hosts used by *P. elegans* in Costa Rica, the possible transmission of this parasite from other non-human primates to squirrel monkeys, and the potential dispersion of the worm to other primate species including humans, should be conducted due to the severe infection produced in its hosts. These analyses are necessary for estimating the impact of human activities in niche and forest fragmentation that may lead to cryptic divergence and eventually parasite speciation.

## Data Availability

Sequences were deposited in GenBank with accession numbers ON458021, ON458022, ON458023, ON458024, ON458025 and ON458026.
